# Metagenomic Profiling of Antibiotic Resistance Genes in Swine Manure Residues After *Sarcophaga peregrina* Larval Bioconversion Under Polystyrene Particle Exposure

**DOI:** 10.3390/insects17090936

**Published:** 2026-09-07

**Authors:** Guanjie Yan, Zhiyuan Ma, Dandan Li, Yuying Li, Zonghua Wang

**Affiliations:** 1 Henan Key Laboratory of Insect Biology in Funiu Mountain, Henan Province Engineering Research Center of Insect Bioreactor, China-UK International Joint Laboratory for Insect Biology of Henan Province, Nanyang Normal University, 1638 Wolong Road, Nanyang 473061, China; guanjiey@gmail.com (G.Y.);; 2 International Joint Laboratory of Watershed Ecological Security for Water Source Region of Mid-line Project of South-to-North Water Diversion in Henan Province, College of South to North Water Diversion/College of Water Resources and Modern Agriculture, Nanyang Normal University, Nanyang 473061, China; lyying200508@163.com

**Keywords:** Sarcophagidae, waste-converting insects, resistome restructuring, CARD annotation, livestock waste recycling, manure-derived residue

## Abstract

Insect-mediated bioconversion is a promising strategy for livestock manure recycling, but its effects on antibiotic resistance genes (ARGs) in residual substrates remain insufficiently understood, especially under polystyrene particle exposure. In this study, swine manure was treated with *Sarcophaga peregrina* larvae under four concentrations of 100-nm polystyrene particles (PS particles: 0, 0.001, 0.01 and 0.1 mg/kg), and ARG profiles in air-dried residual manure were characterized. Larval treatment reduced manure mass by approximately 40% within 4 days, and reduction rates did not differ significantly among PS particle concentrations. However, larval treatment increased RPKM-normalized total ARG abundance relative to the corresponding controls, whereas the residue mass-adjusted relative ARG index remained below 1.0 in all larval treatment groups after incorporating residue mass. ARG richness and Shannon diversity were not significantly reduced. ARO-level turnover analysis showed both newly detected and no longer detected ARG types after larval treatment. PCoA and PERMANOVA indicated treatment-associated differences in resistome structure, with PERMDISP suggesting that dispersion also contributed. At class and mechanism levels, multidrug resistance and antibiotic efflux were dominant. These results indicate that *S. peregrina* larvae efficiently reduce manure mass, but their effects on ARGs are better described as residual resistome restructuring rather than ARG removal.

## Simple Summary

Insect larvae can convert livestock manure into insect biomass and residual substrates, offering a potential strategy for organic waste recycling. However, manure may contain antibiotic resistance genes (ARGs), so larval bioconversion should be evaluated beyond manure mass reduction alone. In this study, *Sarcophaga peregrina* larvae were used to treat swine manure under different 100-nm polystyrene particle concentrations, and ARG profiles in air-dried residual manure were analyzed by metagenomic sequencing. The larvae reduced manure mass by approximately 40% within 4 days, but ARG richness and diversity did not significantly decrease. Larval treatment increased RPKM-normalized ARG abundance and altered resistome composition, whereas PS particle concentration showed weaker effects. After residue mass was considered, the residue mass-adjusted relative ARG index remained below the control level. These findings suggest that ARG-related risks in residual substrates should be independently assessed before recycling or agricultural use.

## 1. Introduction

The rapid expansion of intensive livestock production has generated large amounts of manure that must be properly treated before land application or disposal. Swine manure is not only a nutrient-rich organic resource, but also an important reservoir of antibiotic residues, antibiotic-resistant bacteria and antibiotic resistance genes (ARGs) [1,2,3]. Previous studies have shown that swine farm environments can harbor diverse and abundant ARGs, including genes associated with resistance to clinically important antibiotics and mobile genetic elements that may facilitate horizontal gene transfer [2]. When manure or manure-derived products are returned to agricultural soils, residual ARGs may enter soil–plant–water continuums and contribute to the environmental dissemination of antimicrobial resistance [3]. Therefore, evaluating manure treatment technologies should not be limited to organic matter degradation or nutrient recovery, but should also include the fate of ARGs in the residual substrate.

Biological treatment of animal manure has been widely investigated as a strategy to reduce waste volume and improve resource recovery. Among these approaches, insect-mediated bioconversion has attracted increasing attention because saprophagous larvae can rapidly consume organic wastes and convert them into insect biomass, frass and residual substrates [4]. Black soldier fly larvae have been the most intensively studied insect system, and recent work has examined their effects on antibiotic-resistant bacteria, mobile ARGs and the manure resistome during animal manure treatment [5]. However, the effects of insect bioconversion on ARGs are not necessarily straightforward. Larval feeding, gut passage and microbial succession may jointly alter ARG abundance, richness and composition, and previous studies have reported both ARG reductions in manure residues and ARG retention or accumulation in larval guts during black soldier fly larval bioconversion [5,6,7]. As a result, ARGs in the residual substrate may decrease, persist, become relatively enriched, or undergo compositional shifts depending on the insect species, substrate type and treatment conditions.

The flesh fly *Sarcophaga peregrina* (Robineau-Desvoidy) (synonym *Boettcherisca peregrina*) (Diptera: Sarcophagidae) is a saprophagous dipteran species with potential for organic waste bioconversion. Earlier work showed that *S. peregrina* larvae grown on solid organic wastes, including swine manure, could produce lipid-rich biomass within a short production cycle [8]. More recent studies have further suggested that *S. peregrina* and other manure-associated Diptera species may be useful for swine manure resource utilization and feed-protein production [9]. Nevertheless, compared with black soldier fly larvae, flesh fly larvae have received much less attention in studies of ARG dynamics during livestock manure bioconversion. Assessing ARG abundance and composition in the residual substrate is therefore important for evaluating the environmental safety of *S. peregrina*-mediated manure bioconversion and for linking insect waste-conversion performance with metagenomic indicators of ARG-related risk.

In addition to manure-associated ARGs, microplastics (MPs) represent another class of contaminants that deserves attention in livestock manure management systems. Recent evidence indicates that MPs can occur in livestock manure and manure composting systems, where they may coexist with manure-borne microorganisms, antibiotic-resistant bacteria and ARGs [10]. In manure treatment environments, MPs may provide surfaces for microbial attachment and biofilm formation, thereby creating localized microhabitats in which microbial interactions and gene-transfer processes could occur under suitable conditions. This concern is supported by the plastisphere concept, which describes distinct microbial communities colonizing plastic surfaces, and by experimental evidence showing that microplastic surfaces can increase plasmid-mediated gene transfer in microbial communities [11,12]. Moreover, polystyrene micro- and nanoplastics have been shown to affect biological traits in insects such as *Drosophila melanogaster*, indicating that PS particle exposure may affect insects, although such effects are likely to be species-, particle- and dose-dependent [13]. However, the environmental impacts of low-level PS particle exposure during insect-mediated manure treatment remain poorly understood. Therefore, evaluating potential modifying effects under low-dose exposure scenarios may provide insights into whether background-level plastic particle contamination can influence manure bioconversion-associated ARG dynamics.

In this study, we used a metagenomic approach to evaluate ARG profiles in air-dried residual manure after swine manure bioconversion by *S. peregrina* larvae under four low-dose concentrations of 100-nm polystyrene particles (PS particles). This concentration range was designed as a low-dose exposure gradient to examine whether low-level PS particle exposure could modify larval bioconversion-associated ARG dynamics, rather than to simulate heavily contaminated manure substrates. We aimed to determine: (i) how larval treatment affects manure mass reduction and reads per kilobase per million mapped reads (RPKM)-normalized ARG abundance; (ii) whether PS particle concentration modifies larval effects on ARG richness, diversity and composition; and (iii) how ARG profiles shift at the antibiotic class, resistance mechanism and ARO levels. We further calculated a residue mass-adjusted relative ARG index to jointly consider changes in RPKM-normalized ARG abundance and residual manure mass while recognizing that this index does not represent absolute ARG copy number or absolute ARG load.

## 2. Materials and Methods

### 2.1. Fly Rearing and Manure Preparation

The flesh fly *S. peregrina* used in this study was obtained from a laboratory colony maintained in the insect-breeding laboratory of Nanyang Normal University. The colony was originally established from adults collected on the campus of Nanyang Normal University using manure-baited traps. The colony was maintained under controlled laboratory conditions with a 12:12 h light:dark photoperiod, 30–40% relative humidity and 23–25 °C. Detailed procedures for colony establishment, species identification and continuous laboratory rearing have been described in Yan et al. (2026) [9]. Larvae used in the present experiment were collected from the continuously maintained laboratory colony.

Swine manure was collected from a commercial pig farm in Tanghe County, Nanyang City, Henan Province, China. Fresh manure samples were transported to the laboratory immediately after collection and stored at −20 °C until use. Before the experiment, manure was thawed at room temperature and thoroughly homogenized. Sterile water was added as needed to adjust the moisture content to approximately 75%, and the manure was mixed again to ensure uniform distribution of water and substrate components before allocation to different experimental treatments.

### 2.2. Experimental Design and Sample Collection

**Figure 1:**
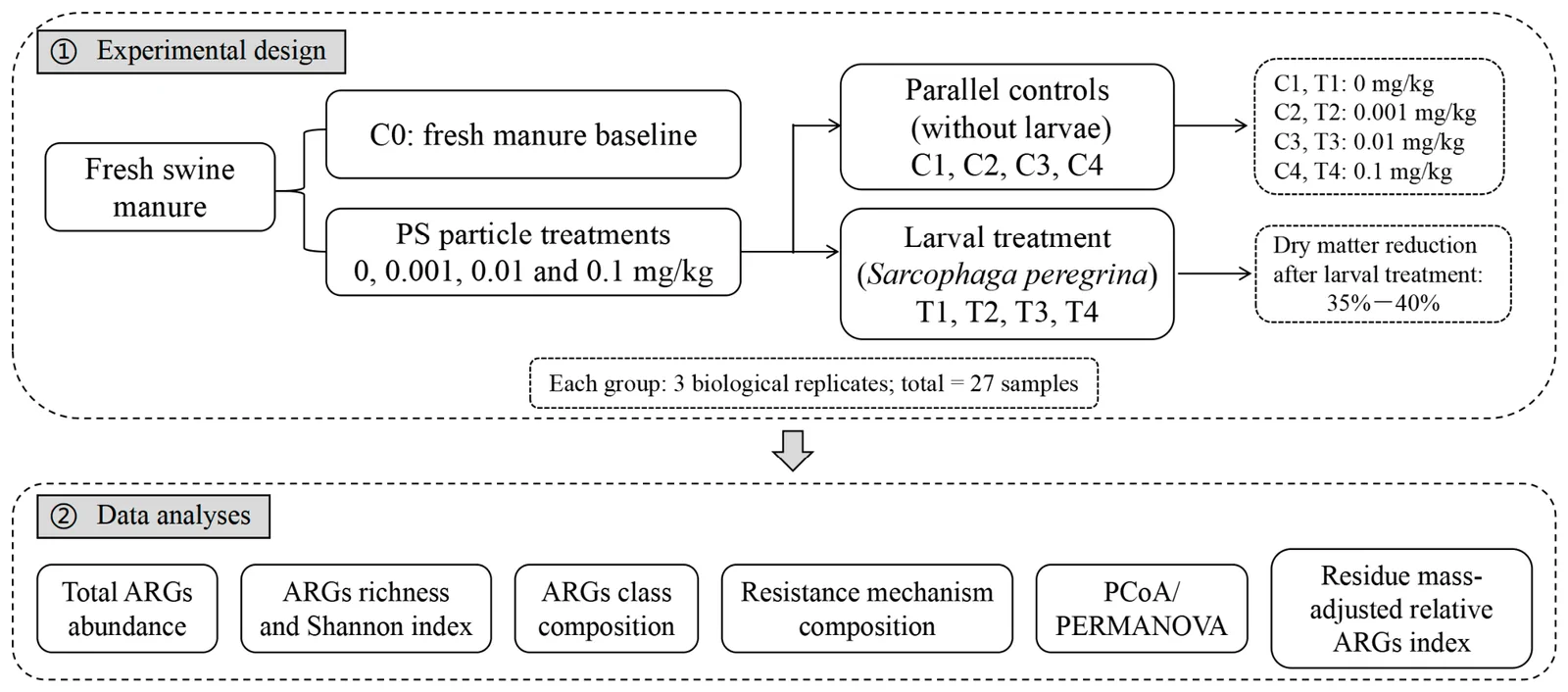
Experimental workflow of swine manure bioconversion by *Sarcophaga peregrina* larvae under 100-nm polystyrene particle exposure. The workflow summarizes manure preparation, PS particle amendment, establishment of the initial manure background group (C0), parallel control groups (C1–C4) and larval treatment groups (T1–T4), 4-day incubation, residue collection and air-drying, metagenomic sequencing, CARD-based ARG annotation and downstream statistical analyses.

This experiment was designed to evaluate the effects of *S. peregrina* larval treatment and 100-nm polystyrene particle exposure on antibiotic resistance genes (ARGs) in swine manure (Figure 1). The initial manure group was designated as C0 and consisted of prepared manure collected before larval incubation. This group was used to represent the initial ARG background.

Prepared swine manure was amended with polystyrene particles (PS particles; nominal diameter: 100 nm; Tianjin Base Line Chrom Tech Research Centre, Tianjin, China) to obtain final concentrations of 0, 0.001, 0.01 and 0.1 mg/kg wet manure substrate. Before addition to the manure substrate, the PS particle stock suspension was thoroughly mixed and diluted to the required working concentrations. The diluted PS particle suspensions were then manually mixed throughout the prepared manure substrate to promote particle dispersion. The 0 mg/kg groups received the same volume of particle-free sterile water and were mixed in the same manner. The exposure concentrations were selected based on previous insect studies using low-dose PS particle exposure gradients [13] and were adapted here to establish a 10-fold low-dose concentration gradient suitable for a manure bioconversion system, rather than to simulate heavily contaminated manure substrates. For each PS particle concentration, two treatments were established: a parallel control without larvae and a larval treatment inoculated with *S. peregrina* larvae. The parallel control groups were designated as C1, C2, C3 and C4, and the larval treatment groups were designated as T1, T2, T3 and T4. C1/T1, C2/T2, C3/T3 and C4/T4 corresponded to 0, 0.001, 0.01 and 0.1 mg/kg PS particle treatments, respectively. All treatment units were incubated under the same laboratory conditions for 4 days. Together with the initial manure background group C0, each group contained three biological replicates, resulting in a total of 27 metagenomic samples.

For each biological replicate, 300 g of prepared manure substrate amended with the corresponding PS particle concentration was placed into a 750 mL disposable plastic container (bottom diameter: 10.5 cm). In the larval treatment groups, 300 S. peregrina larvae were introduced into each container, whereas the corresponding parallel control groups received the same amount of substrate but no larvae. This inoculation density corresponded to 1 larva/g wet substrate, or approximately 4 larvae/g initial dry matter. It was selected based on our preliminary experiments, in which larvae reared at this substrate-to-larva ratio reached more than 90% of the body weight observed under the reference rearing condition in the preliminary experiment. Therefore, this density was considered suitable for maintaining larval growth and manure bioconversion under controlled laboratory conditions, rather than representing a direct engineering-scale application rate. Three biological replicates were established for each treatment. All treatments and biological replicates were prepared from the same homogenized manure batch to minimize initial substrate heterogeneity. Therefore, the biological replicates should be interpreted as independent experimental containers rather than independent manure collections, with each container considered an independent experimental unit. During the 4-day incubation period, the substrate in both larval treatment and parallel control units was gently mixed once daily from day 2 to day 4 to maintain substrate homogeneity and to ensure comparable disturbance between treatments. No additional substrate was added during the incubation period. At the end of the experiment, visible larvae were removed from the larval treatment units before residue processing. The remaining manure residues were naturally air-dried under identical laboratory conditions before weighing. The parallel control residues were processed using the same air-drying and weighing procedure. Air drying was applied to standardize residual manure samples for residue mass determination and downstream DNA extraction by reducing variation caused by differences in water content among samples. Because the manure moisture content was adjusted to approximately 75%, the initial dry manure mass was calculated from the initial wet manure mass and the corresponding dry matter fraction. The reduction rate of manure dry matter was calculated as follows:

Manure reduction rate (%) = [(initial dry manure mass − air-dried residue mass)/initial dry manure mass] × 100

After weighing, air-dried residual manure samples were thoroughly homogenized, and subsamples were collected for metagenomic sequencing. Therefore, ARG profiles in this study represent the DNA-based ARG composition and RPKM-normalized ARG abundance of air-dried residual manure rather than those of fresh wet manure immediately after incubation or absolute ARG copy numbers.

### 2.3. Metagenomic Sequencing and ARG Profiling

Total DNA was extracted from manure samples using the E.Z.N.A.^®^ Soil DNA Kit (Omega Bio-tek, Norcross, GA, USA) according to the manufacturer’s instructions. DNA concentration and purity were assessed using a NanoDrop 2000 spectrophotometer (Thermo Fisher Scientific, Wilmington, DE, USA), and DNA integrity was checked by 1% agarose gel electrophoresis. Qualified DNA was sheared into fragments of approximately 400 bp using a Covaris M220 system (Covaris, LLC, Woburn, MA, USA). Paired-end sequencing libraries were prepared using the NEXTFLEX^TM^ Rapid DNA-Seq Kit (Bioo Scientific Corporation, Austin, TX, USA) following the manufacturer’s protocol. Libraries were constructed with an insert size of approximately 500 bp and sequenced on an Illumina NovaSeq platform using a paired-end 150-bp sequencing strategy.

Raw reads were separated according to sample-specific index sequences and stored in FASTQ format. Quality control was performed using fastp v0.20.0 [14] to remove adaptor-contaminated reads, low-quality reads and reads containing ambiguous bases. The resulting high-quality reads were used for downstream assembly, gene prediction and functional annotation.

Clean reads from each sample were assembled using MEGAHIT v1.1.2 [15]. Open reading frames were predicted from assembled contigs using Prodigal v2.6.3 [16], and predicted genes with nucleotide lengths of at least 100 bp were retained and translated into amino acid sequences. Predicted genes from all samples were pooled and clustered using CD-HIT v4.6.1 [17]. The longest sequence in each cluster was selected as the representative sequence to construct a non-redundant gene catalogue. Raw and clean sequencing statistics, assembly statistics, gene prediction statistics and non-redundant gene catalogue information are summarized in Table S1.

### 2.4. CARD Annotation and ARO-Level Abundance Calculation

To estimate gene abundance, high-quality reads from each sample were mapped back to the non-redundant gene catalogue using SOAPaligner v2.21 [18]. Gene-level abundance profiles were generated as reads number, RPKM and TPM matrices. RPKM values were used as the primary abundance metric in this study because they account for both sequencing depth and gene length, whereas reads number was retained for presence/absence filtering and consistency checks.

ARG annotation was performed by comparing the predicted protein sequences of the non-redundant gene catalogue against the Comprehensive Antibiotic Resistance Database, CARD v3.0.9 [19], using DIAMOND v0.8.35 [20]. ARGs were summarized at the ARO accession level. Gene-to-ARO mapping information was obtained from the CARD ARO gene number table, which lists the non-redundant genes assigned to each ARO entry. Gene-level reads number, RPKM and TPM values were then summed by ARO accession to generate ARO-level abundance matrices. Annotation fields, including ARO name, AMR gene family, drug class, antibiotic class and resistance mechanism, were merged into the final ARO abundance table.

Detailed CARD alignment information, including alignment score, E-value, sequence identity, similarity, alignment length and alignment coordinates, was retained for downstream ARG analyses. The retained CARD alignment results had E-values ≤ 1 × 10^−5^. Query coverage and hit coverage were calculated based on the query and hit alignment coordinates. CARD annotation and alignment information is summarized in Table S2, and the full CARD alignment and annotation results are provided in Supplementary Data S1.

### 2.5. ARG Filtering and Statistical Analysis

Rare and poorly reproducible AROs were removed before downstream analyses. AROs were retained if they were detected in at least two of three biological replicates within at least one treatment group and had a total read count ≥ 100 across all samples. Detection was defined as read count > 0. The filtered ARO-level RPKM matrix was used for abundance-based analyses, including total ARG abundance, Shannon diversity, antibiotic class composition, resistance mechanism composition and resistome structure. The filtered ARO read-count matrix was used for detection-based analyses, including ARG richness and ARO turnover. ARG richness was calculated as the number of retained AROs detected in each sample.

ARO turnover between larval treatment groups and their corresponding parallel controls was evaluated at the group level. An ARO was considered detected in a group if it had read count > 0 in at least two of the three biological replicates. For each T/C comparison at the same PS particle concentration, shared AROs were defined as those detected in both the larval treatment group and the corresponding control group. Newly detected AROs were defined as AROs detected only in the larval treatment group, whereas no longer detected AROs were defined as AROs detected only in the corresponding control group. Net change was calculated as the number of newly detected AROs minus the number of no longer detected AROs.

Total ARG abundance was calculated for each sample by summing the RPKM values of all retained AROs. The T/C fold change was calculated for each larval treatment replicate as the ratio of total ARG RPKM in the larval residue to the mean total ARG RPKM of the corresponding control group at the same PS particle concentration. This index reflects the relative change in RPKM-normalized ARG abundance in larval residues without considering residual manure mass reduction.

For antibiotic class and resistance mechanism analyses, RPKM values of AROs assigned to the same antibiotic class or resistance mechanism were summed within each sample. Relative abundance was calculated as the proportion of each category in the total RPKM-normalized ARG abundance of each sample, and low-abundance categories were grouped as “Others” for visualization. For two-way ANOVA of antibiotic class and resistance mechanism abundance, category-level summed RPKM values were log10-transformed before analysis. To account for multiple category-level tests, Benjamini–Hochberg false discovery rate (FDR) correction was applied separately to antibiotic class and resistance mechanism results within each model factor, including larval treatment, PS particle concentration and their interaction. Categories with raw *p* < 0.05 but FDR-adjusted *p* ≥ 0.05 were interpreted as nominally significant only.

Because larval treatment reduced residual manure mass, a residue mass-adjusted relative ARG index was calculated to integrate RPKM-normalized ARG abundance with residual manure mass. For each larval treatment replicate, the index was calculated by multiplying the T/C fold change in total ARG RPKM by the ratio of the air-dried residue mass of that larval replicate to the mean air-dried residue mass of the corresponding parallel control group at the same PS particle concentration. The corresponding control level was set to 1.0. This index was used as a relative metric and should not be interpreted as an absolute ARG copy number or absolute ARG load.

Bray–Curtis dissimilarity was used to assess differences in ARG composition among samples, and principal coordinate analysis (PCoA) was performed to visualize resistome variation. PERMANOVA with 999 permutations [21] was first used to compare ARG composition among fresh manure, parallel control and larval treatment samples. After excluding the initial manure group C0, two-factor PERMANOVA was further performed to test the effects of larval treatment, PS particle concentration and their interaction on overall ARG composition. The initial manure group C0 was used only as a background reference and was not included in factorial statistical analyses. To evaluate whether PERMANOVA results were potentially influenced by differences in within-group dispersion, PERMDISP tests based on Bray–Curtis dissimilarities were performed, and distances to group centroids were tested using 999 permutations.

For C1–C4 and T1–T4, two-way ANOVA was used to test the effects of larval treatment, PS particle concentration and their interaction on total ARG abundance, ARG richness, Shannon diversity and major ARG categories. Manure reduction rates among T1–T4 were compared using one-way ANOVA. Data were log10-transformed when necessary to improve normality and homogeneity of variance. Statistical significance was set at *p* < 0.05.

## 3. Results

### 3.1. Sequencing Output, CARD Annotation and ARO Filtering

Sequencing quality, assembly statistics, gene prediction statistics and non-redundant gene catalogue characteristics are summarized in Table S1. A non-redundant gene catalogue containing 1,794,793 genes was constructed and used for downstream abundance estimation. The CARD annotation and alignment summary is provided in Table S2, and the full CARD alignment and annotation details are provided in Supplementary Data S1.

The unfiltered CARD annotation dataset contained 805 ARO entries across all samples. To reduce the influence of rare AROs and low-support detections, AROs were retained only when they were detected in at least two of three biological replicates within at least one treatment group and had a total read count of at least 100 across all samples. After filtering, 692 AROs were retained for downstream analyses, whereas 113 rare or low-support AROs were removed. Detailed information on ARO filtering and group-level ARG metrics is summarized in Table S3. The filtered ARO-level RPKM matrix was used to evaluate total ARG abundance, Shannon diversity, antibiotic class composition, resistance mechanism composition and ARG compositional variation, whereas the filtered reads matrix was used for detection-based analyses, including ARG richness and ARO turnover. This filtering strategy was used to reduce the influence of sporadic detections while retaining AROs with sufficient reproducibility and read support for comparative analyses.

### 3.2. Manure Reduction and Total ARG Abundance in Air-Dried Residues

**Figure 2:**
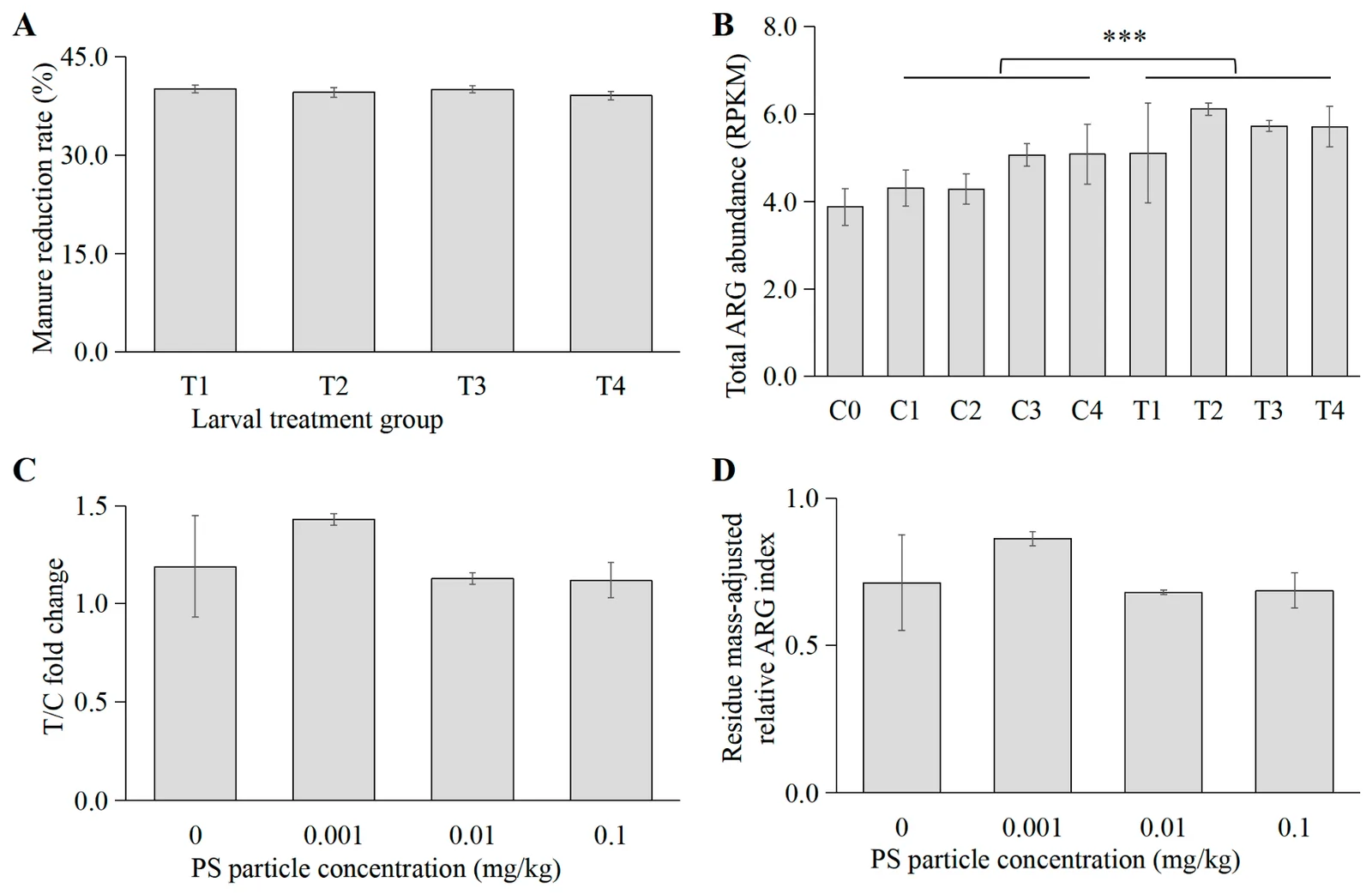
Manure reduction and RPKM-normalized total ARG abundance in air-dried residues. Data are shown as mean ± SD (*n* = 3 biological replicates). (**A**) Manure reduction rates in larval treatment groups after 4 days of bioconversion. (**B**) Total ARG abundance based on the filtered ARO-level RPKM matrix in C0, C1–C4 and T1–T4. (**C**) Fold change in total ARG abundance between each larval treatment group and its corresponding control group. (**D**) Residue mass-adjusted relative ARG index. For panel B, two-way ANOVA was performed on log10-transformed total ARG abundance after excluding C0, and *** indicates a significant main effect of larval treatment (*p* < 0.001). The index in (**D**) was calculated as the T/C fold change in total ARG RPKM multiplied by the ratio of larval residue mass to the mean residue mass of the corresponding parallel control group. This relative index does not represent absolute ARG copy number or absolute ARG load.

*S. peregrina* larvae substantially reduced manure mass during the 4-day treatment period (Figure 2A). The manure reduction rates were 40.08 ± 0.56%, 39.55 ± 0.75%, 39.99 ± 0.53% and 39.05 ± 0.63% in T1, T2, T3 and T4, respectively. No significant difference in manure reduction rate was detected among the four PS particle concentrations (one-way ANOVA, F_3,8_ = 1.74, *p* = 0.24).

In contrast, larval treatment was associated with higher RPKM-normalized total ARG abundance in the air-dried residues relative to the corresponding parallel controls, with T/C fold changes above 1.0 at all PS particle concentrations (Figure 2B,C). Two-way ANOVA showed a significant effect of larval treatment on total ARG abundance (F_1,16_ = 16.77, *p* < 0.001), whereas PS particle concentration (F_3,16_ = 2.23, *p* = 0.12) and the larval treatment × PS particle concentration interaction (F_3,16_ = 1.50, *p* = 0.25) were not significant (Table S4). The T/C fold changes were 1.19, 1.43, 1.13 and 1.12 for T1/C1, T2/C2, T3/C3 and T4/C4, respectively (Figure 2C).

A residue mass-adjusted relative ARG index was further calculated by incorporating both RPKM-normalized total ARG abundance and the measured residue mass of each larval replicate relative to the mean residue mass of the corresponding parallel control group. The adjusted indices were 0.712 ± 0.163, 0.863 ± 0.024, 0.680 ± 0.009 and 0.686 ± 0.061 for T1, T2, T3 and T4, respectively, with all mean values below the normalized control reference value of 1.0 (Figure 2D). Because this index was derived from RPKM-normalized abundance and relative residue mass, it should be interpreted as a relative estimate rather than as a direct measurement of absolute ARG copy number or absolute ARG load.

### 3.3. ARG Richness, Diversity and Detection-Based ARO Turnover

**Figure 3:**
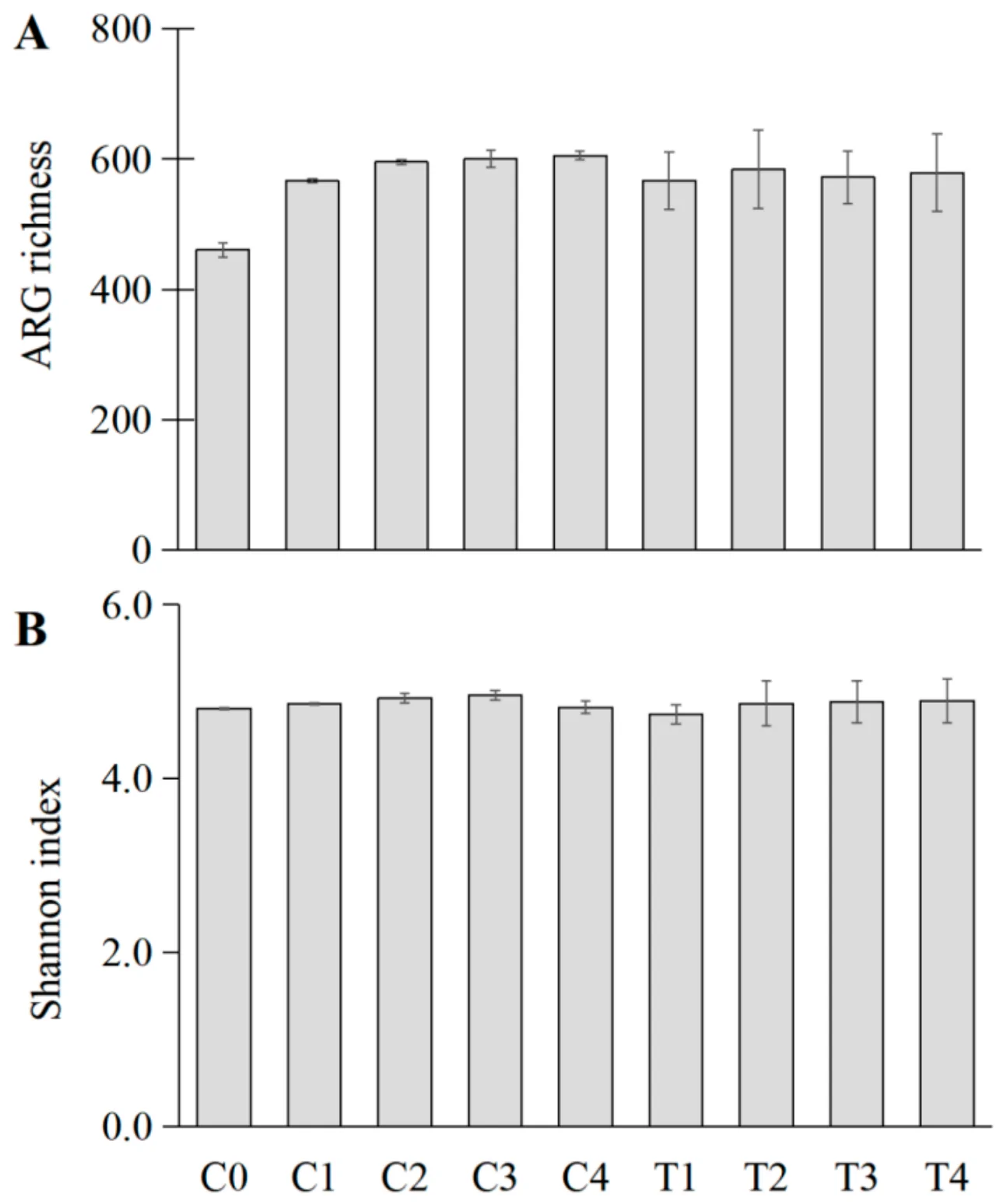
ARG richness and Shannon diversity in air-dried residual manure. (**A**) ARG richness represents the number of detected retained AROs based on the filtered ARO read-count matrix. (**B**) Shannon diversity was calculated using the RPKM-normalized abundance of retained AROs. Bars represent mean ± SD, *n* = 3. C0 represents the initial manure group. C1–C4 represent parallel control groups without larvae, and T1–T4 represent larval treatment groups. C1/T1, C2/T2, C3/T3 and C4/T4 correspond to 0, 0.001, 0.01 and 0.1 mg/kg PS particles, respectively.

ARG richness was calculated as the number of detected retained AROs, whereas Shannon diversity was calculated using the filtered ARO-level RPKM matrix (Figure 3A,B). After excluding the initial manure group C0, two-way ANOVA showed that ARG richness was not significantly affected by larval treatment (F_1,16_ = 1.21, *p* = 0.287), PS particle concentration (F_3,16_ = 0.61, *p* = 0.621), or their interaction (F_3,16_ = 0.20, *p* = 0.898). Similarly, no significant effects of larval treatment (F_1,16_ = 0.42, *p* = 0.526), PS particle concentration (F_3,16_ = 0.64, *p* = 0.599), or their interaction (F_3,16_ = 0.42, *p* = 0.742) were detected for Shannon diversity (Table S4).

At the group level, larval treatment groups showed both newly detected and no longer detected AROs relative to the corresponding controls (Table S5). These turnover patterns are further visualized in Figure S1, which summarizes the numbers of shared, newly detected and no longer detected AROs for each larval treatment group relative to its corresponding parallel control. Because detection-based turnover can be influenced by low-abundance AROs near the detection threshold, these terms were used operationally and should not be interpreted as definitive acquisition or loss of ARGs. Detailed ARO-level turnover information, including mean read counts and RPKM values in corresponding control and larval treatment groups, is provided in Supplementary Data S2.

### 3.4. ARG Class, Resistance Mechanism and Compositional Variation

**Figure 4:**
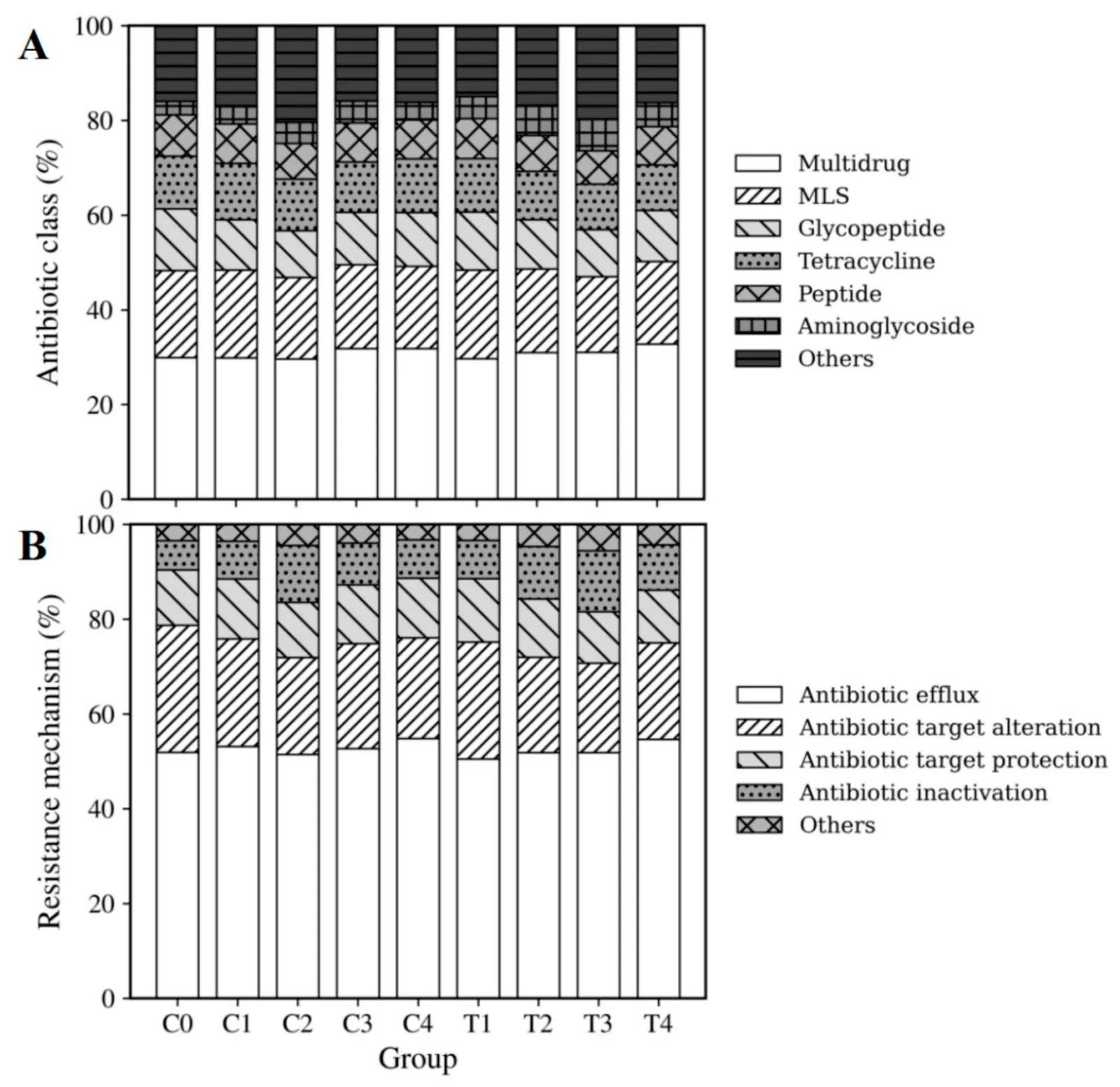
Composition of ARG antibiotic classes and resistance mechanisms in air-dried residual manure. (**A**) Relative abundance of ARG antibiotic classes based on summed RPKM values of AROs assigned to each class. (**B**) Relative abundance of ARG resistance mechanisms based on summed RPKM values of AROs assigned to each mechanism. Low-abundance categories were grouped as “Others” for visualization. C0 represents the initial manure group. C1–C4 represent parallel control groups without larvae, and T1–T4 represent larval treatment groups. C1/T1, C2/T2, C3/T3 and C4/T4 correspond to 0, 0.001, 0.01 and 0.1 mg/kg PS particles, respectively. Statistical analyses of category-level summed RPKM values were performed using two-way ANOVA followed by Benjamini–Hochberg FDR correction, and the results are provided in Table S4.

The ARG profiles were dominated by Multidrug, MLS, Glycopeptide, Tetracycline and Peptide resistance classes, and by antibiotic efflux, target alteration, target protection and antibiotic inactivation mechanisms (Figure 4). After FDR correction, larval treatment remained significantly associated with several ARG classes, including Multidrug, MLS, Glycopeptide, Peptide, Aminoglycoside, Mupirocin and Pleuromutilin, and with several resistance mechanisms, including antibiotic efflux, antibiotic inactivation and antibiotic efflux combined with reduced permeability. In contrast, PS particle concentration showed nominal effects on several ARG classes and resistance mechanisms before FDR correction, but none of these effects remained significant after FDR correction. No interaction effect remained significant after FDR correction (Table S4).

**Figure 5:**
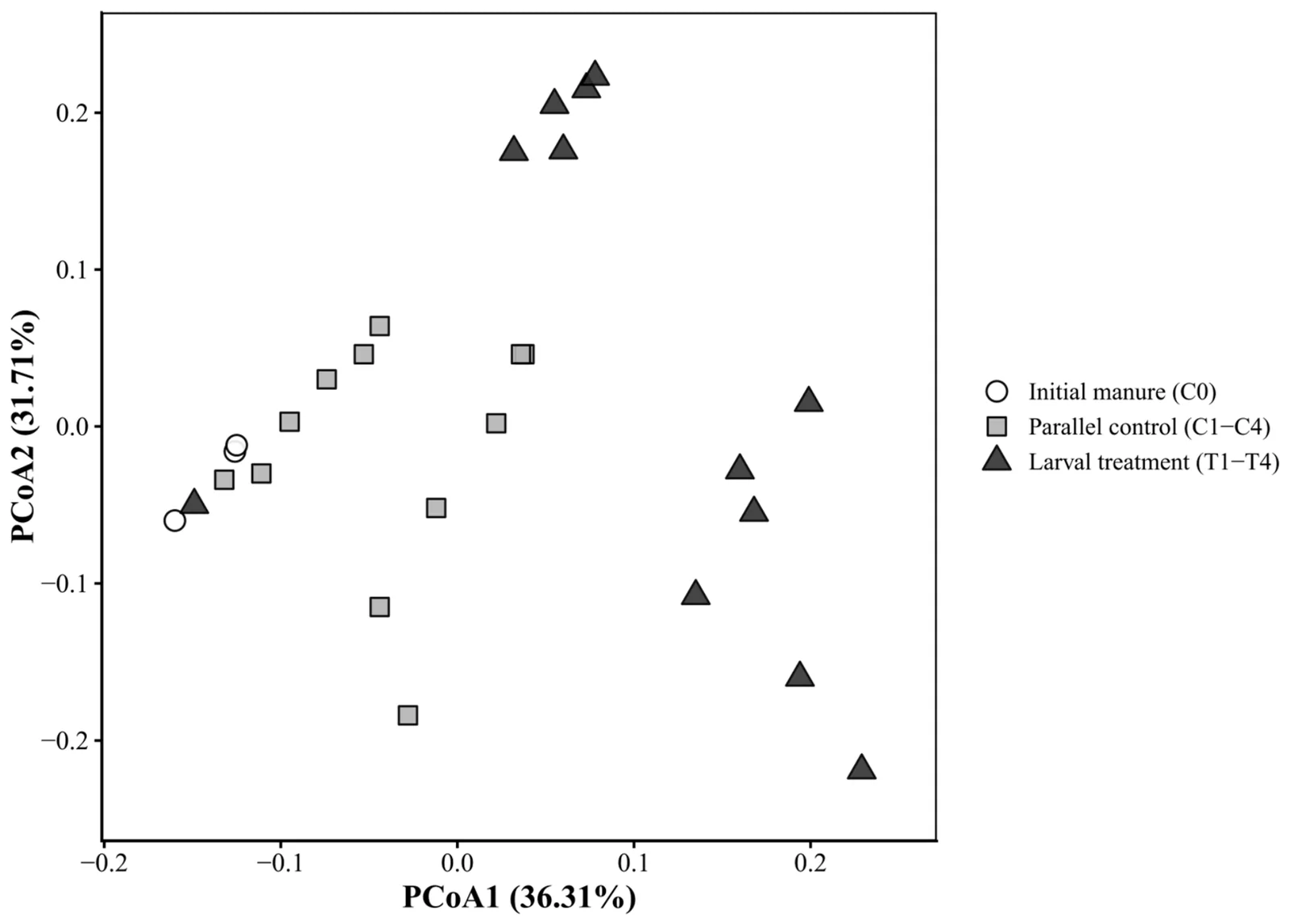
Principal coordinate analysis (PCoA) of ARG composition based on Bray–Curtis dissimilarities of the filtered ARO-level RPKM matrix. Each point represents one biological replicate. PCoA1 and PCoA2 explained 36.31% and 31.71% of the total variation, respectively. Initial manure (C0) represents the initial manure background group, parallel controls (C1–C4) represent non-larval manure residues, and larval treatment groups (T1–T4) represent manure residues after *Sarcophaga peregrina* larval bioconversion. PERMANOVA and PERMDISP results are provided in Table S4.

PCoA based on Bray–Curtis dissimilarity of the filtered ARO-level RPKM matrix showed treatment-associated variation in ARG composition (Figure 5). PERMANOVA showed significant differences in ARG composition among initial manure background, parallel control and larval treatment samples (R^2^ = 0.313, F = 5.471, *p* = 0.001). After excluding the initial manure group C0, two-factor PERMANOVA further showed that larval treatment was significantly associated with ARG composition variation (R^2^ = 0.237, F = 8.004, *p* = 0.001), whereas PS particle concentration (R^2^ = 0.154, F = 1.732, *p* = 0.083) and the larval treatment × PS particle concentration interaction (R^2^ = 0.136, F = 1.538, *p* = 0.139) were not significant.

PERMDISP tests indicated significant differences in within-group dispersion among treatment types and between control and larval treatment samples (Table S4). Therefore, PERMANOVA results were interpreted as evidence of treatment-associated differences in overall multivariate ARG structure, potentially involving both centroid shifts and dispersion differences.

## 4. Discussion

### 4.1. Manure Mass Reduction

*S. peregrina* larvae reduced swine manure mass by approximately 40% within 4 days, showing a clear capacity for rapid substrate conversion under laboratory conditions. This agrees with previous work showing that fly larvae can transform organic wastes into larval biomass and residual substrates, and supports the potential use of manure-associated Diptera in organic waste bioconversion systems [4]. Earlier studies have also reported that *S. peregrina* can develop on solid organic wastes, including swine manure, and produce lipid- or protein-rich biomass within a short production cycle [8,9]. Thus, although black soldier fly larvae remain the best-studied model for insect-mediated waste treatment, flesh fly larvae may also be useful for swine manure resource utilization.

Manure reduction rates were similar across the four PS particle concentrations, indicating that the exposure levels used here did not markedly impair the substrate-reducing performance of *S. peregrina* larvae. This point is important because differences in ARG profiles among larval treatment groups were unlikely to be explained by large differences in manure reduction performance. However, substrate reduction is only one aspect of treatment performance. Studies on black soldier fly larval conversion and post-conversion composting have emphasized that residual products should also be evaluated for ARGs, antibiotic-resistant bacteria and antibiotic-resistant pathogenic bacteria [5]. In addition, insect-derived frass applied to cropland has been examined as a potential route for transferring resistance genes to rhizosphere soil and plant-associated microorganisms [22]. More broadly, the fate of ARGs during manure treatment is known to be shaped by microbial succession, mobile genetic elements and environmental conditions, rather than by organic matter degradation alone [23]. These studies support the view that environmental evaluation of *S. peregrina*-mediated manure bioconversion should integrate both manure mass reduction and molecular indicators of residual ARG-related risk.

### 4.2. Effects of Larval Treatment and PS Particles on Residual ARG Profiles

Compared with PS particle concentration, larval treatment was the stronger factor shaping residual ARG profiles. This is consistent with previous studies showing that insect-mediated manure bioconversion can alter manure-associated resistomes through larval feeding, gut passage and microbially mediated substrate transformation [5,6,7]. In addition, recent studies on *S. peregrina* have shown that this flesh fly can efficiently utilize swine manure for biomass production and that its internal or gut-associated microbiota changes during development on manure substrates [9,24]. In the present short-term system, PS particle concentration did not significantly affect total ARG abundance, ARG richness, Shannon diversity or overall ARG composition. After FDR correction, PS particle concentration also did not retain significant effects on ARG classes or resistance mechanisms, although several categories showed nominal responses before correction. Similar patterns have been reported in manure composting systems, where microplastics did not necessarily cause broad changes in total ARG composition or abundance, but may still be associated with changes in specific ARG hosts or gene-transfer risks under certain conditions [25]. Reviews on livestock manure and composting systems also suggest that MPs may influence ARG dissemination by altering microbial microhabitats, selective pressure, oxidative stress or membrane permeability [10]. A recent study on *S. peregrina* further showed that PS particles can be ingested by larvae and can interact with host-associated microbiota under livestock-manure-related rearing conditions, supporting the need to consider plastic-particle-associated risks in waste-to-protein bioconversion systems [26]. In our study, however, these potential mechanisms should be interpreted cautiously because PS particle concentration did not show robust effects after FDR correction. The relatively weak influence of PS particles may also be related to the low exposure concentrations applied in this study, which were designed as a low-dose exposure gradient and were not intended to represent heavily contaminated manure environments. Thus, the effect of PS particles in this low-dose exposure system should be interpreted as limited and potentially category-specific, rather than as a dominant driver of the residual resistome.

The effect of larval treatment on ARGs was better described as resistome restructuring rather than ARG removal. Although *S. peregrina* larvae markedly reduced manure mass, ARG richness and Shannon diversity did not decrease significantly, and the larval treatment groups contained both newly detected and no longer detected AROs relative to their corresponding controls. It should be noted that the initial manure group (C0) and the parallel control groups (C1–C4) represented different incubation stages, and the differences between these groups suggest that background processes such as microbial succession and substrate handling could also contribute to resistome variation. Therefore, the effects attributed to larval treatment in this study represent changes relative to the corresponding time-matched parallel controls rather than changes directly from the initial manure state. Together with the PERMANOVA results, this suggests that larval bioconversion changed the overall multivariate structure of the residual resistome without uniformly eliminating ARG types. However, because PERMDISP tests were significant, these PERMANOVA results should be interpreted cautiously, as the observed differences may reflect both shifts in group centroids and changes in within-group dispersion. This type of response is plausible because ARG dynamics during insect-mediated manure conversion can vary with substrate type, larval gut-associated microbiota, microbial succession in the residue and subsequent stabilization processes [5,6,7,24].

The higher RPKM-normalized total ARG abundance in larval residues also needs careful interpretation. RPKM values are useful for comparing relative metagenomic abundance after correction for sequencing depth and gene length, but they do not represent absolute ARG copy numbers per unit mass; absolute quantification generally requires qPCR, ddPCR or quantitative metagenomic approaches with internal standards [27,28]. Therefore, the higher RPKM-normalized total ARG abundance in larval residues should be interpreted as relative enrichment rather than an increase in ARG copy number. This relative enrichment may result from several non-exclusive processes. First, rapid degradation of organic matter and reduction of residual manure mass may increase the relative representation of ARG-carrying microbial DNA in the remaining residue. Second, larval feeding and gut passage may selectively affect manure-associated microbial populations, as suggested by previous studies on manure-converting insects and by recent work showing development-associated internal microbiota shifts in *S. peregrina* reared on swine manure [5,6,7,24]. Third, microbial succession during residue stabilization may alter the relative contribution of ARG-carrying taxa to the residual metagenome. However, because larval gut microbiota, larval biomass-associated ARGs and host-resolved ARG profiles were not analyzed in the present study, these explanations should be considered plausible mechanisms rather than demonstrated pathways. When residue mass was incorporated relative to the corresponding controls, the residue mass-adjusted relative ARG index remained below 1.0 across all larval treatment groups. Thus, larval treatment reduced residual manure mass while restructuring the residual resistome, rather than simply increasing or eliminating ARGs.

### 4.3. ARG Class and Resistance Mechanism Responses

The class- and mechanism-level results further indicate that larval bioconversion reshaped specific functional components of the residual resistome. Multidrug, MLS, Glycopeptide, Tetracycline and Peptide resistance categories accounted for a major fraction of ARG abundance, while antibiotic efflux was the dominant resistance mechanism. Such patterns are consistent with the complex resistome background commonly reported in livestock manure, where diverse ARGs, multidrug resistance determinants and mobile genetic elements frequently coexist [2,27]. Metagenomic studies have also shown that multidrug ARGs and efflux pump-associated genes are important components of manure-associated resistomes and may be linked to potential bacterial hosts such as members of Enterobacteriaceae [29]. In swine manure, resistome profiles can differ at both antimicrobial class and resistance mechanism levels among production systems, indicating that these functional profiles are sensitive to microbial community background, management practices and selective pressures [30]. Therefore, the dominance of multidrug- and efflux-related categories in this study most likely reflects the broad resistance reservoir of swine manure rather than the behavior of a single resistance subtype.

After FDR correction, several ARG classes and resistance mechanisms remained significantly associated with larval treatment, whereas others were less responsive. This selective pattern is important because it shows that larval bioconversion did not uniformly increase or decrease all ARG categories. Instead, it shifted the functional structure of the residual resistome, possibly through changes in microbial hosts, mobile genetic elements, co-selection pressures or residue microenvironments, which are known to influence ARG fate during manure treatment [5,23,29]. By contrast, PS particle concentration showed only nominal category-level effects before FDR correction, and these effects did not remain significant after FDR correction. Therefore, PS particles should be interpreted as a potential weak modifier under the present low-dose exposure conditions, rather than as a dominant driver of the overall functional resistome. Because ARGs were not directly linked to bacterial hosts, plasmids or mobile genetic elements in this study, the class- and mechanism-level changes should be interpreted as evidence of functional restructuring, while the microbial carriers and transfer potential behind these shifts require host-resolved metagenomic analysis.

### 4.4. Implications, Limitations and Future Work

This study provides a metagenomic assessment of ARG-related risks during *S. peregrina*-mediated swine manure bioconversion under PS particle exposure. The results show that the residual ARG profile did not change in parallel with manure reduction. This finding is relevant for insect-based manure treatment systems because residual manure, frass-like materials or post-treatment products may enter composting, storage or agricultural recycling processes. Similar concerns have been raised in studies on insect-derived residues, manure treatment systems and manure/compost-associated microplastic contamination, where residual ARGs, antibiotic-resistant bacteria and potential ARG transmission risks remain important considerations [5,10,22].

From an application perspective, these results indicate that *S. peregrina*-based manure bioconversion should not be evaluated solely by manure mass reduction. Because larval treatment restructured the residual resistome and increased RPKM-normalized total ARG abundance, residual manure should be considered for further stabilization or composting, and ARG risk assessment should be conducted before land application [5,22]. In addition, because ARGs in larval guts and larval biomass were not analyzed in this study, larval biomass should be evaluated separately for ARG carriage and microbial safety before use as animal feed or other recycling products. These precautions are particularly important when manure substrates contain plastic particle contamination or other co-selective stressors [10].

These findings should be interpreted with appropriate caution. ARG abundance was evaluated using RPKM-normalized metagenomic data, which allow comparison of resistome profiles among treatments but do not provide absolute ARG copy numbers. In addition, the metagenomic profiles were obtained from air-dried residual manure, and therefore represent DNA-based ARG composition and normalized ARG abundance in air-dried residues rather than viable ARG hosts or absolute ARG loads in fresh manure. Because air-drying may cause microbial cell lysis or selective loss of viable cells, the ARG profiles reported here may deviate from the viable microbial community and ARG–host structure present in fresh wet residues. This study did not annotate mobile genetic elements or directly assess horizontal gene transfer potential. ARGs in larval guts or larval biomass were also not analyzed. Therefore, the present data cannot distinguish whether ARG changes in residual manure were associated with larval gut passage, ARG transfer into larval biomass or residue-associated microbial succession. The significant PERMDISP results further indicate that multivariate ARG compositional differences should be interpreted as overall structural differences that may include both centroid shifts and dispersion effects. Future studies should combine metagenomic profiling with MGE annotation, host-resolved metagenomics, qPCR, ddPCR or quantitative metagenomics, and paired analyses of manure residues and larval tissues to verify absolute changes in key ARGs and clarify ARG transfer pathways. Such work would provide a stronger basis for assessing the biosafety of *S. peregrina*-based manure bioconversion systems.

## Data Availability

The raw metagenomic sequencing data generated in this study have been deposited in the NCBI Sequence Read Archive (SRA) under accession numbers SRR26830820–SRR26830846. The processed ARO-level abundance matrices, filtering results, statistical outputs and source data used to generate the figures and Supplementary tables are provided in the Supplementary Materials.

## Supplementary Materials

The following supporting information can be downloaded at: https://www.mdpi.com/article/10.3390/insects17090936/s1, Table S1: Summary of sequencing quality, assembly, gene prediction and non-redundant gene catalogue statistics; Table S2: Summary of CARD annotation and alignment information; Table S3: Summary of CARD annotation, ARO filtering and group-level ARG metrics; Table S4: Statistical analyses for ARG metrics and resistome composition; Table S5: Group-level ARO turnover summary; Supplementary Data S1: Full CARD alignment and annotation details; Supplementary Data S2: ARO-level turnover details. Figure S1: Visualization of ARO turnover patterns between larval treatment groups and corresponding parallel controls.
