# Supplementary material for: Metagenomic Profiling of Antibiotic Resistance Genes in Swine Manure Residues After Sarcophaga peregrina Larval Bioconversion Under Polystyrene Particle Exposure
Source: Insects. 2026 Sep 7;17(9):936. doi: 10.3390/insects17090936 (PMC13607703; doi:10.3390/insects17090936)
Supplement: Supplementary file 1 [file insects-17-00936-s001.zip › Supplementary Table S5.pdf]

Table S5. Group-level ARO turnover summary

Control group	Larval group	PS particles (mg/kg)	Control detected AROs	Larval detected AROs	Shared AROs	Newly detected in larval group	No longer detected in larval group	Net change
C1	T1	0	567	575	534	41	33	+8
C2	T2	0.001	603	605	555	50	48	+2
C3	T3	0.01	607	603	558	45	49	-4
C4	T4	0.1	609	592	546	46	63	-17

Note: "Newly detected" and "no longer detected" are operational detection-based terms and should not be interpreted as definitive ARG acquisition or loss. Detailed ARO-level turnover information is provided in Supplementary Data S2.
