# Supplementary material for: Metagenomic Profiling of Antibiotic Resistance Genes in Swine Manure Residues After Sarcophaga peregrina Larval Bioconversion Under Polystyrene Particle Exposure
Source: Insects. 2026 Sep 7;17(9):936. doi: 10.3390/insects17090936 (PMC13607703; doi:10.3390/insects17090936)
Supplement: Supplementary file 1 [file insects-17-00936-s001.zip › Supplementary Figure S1.pdf]

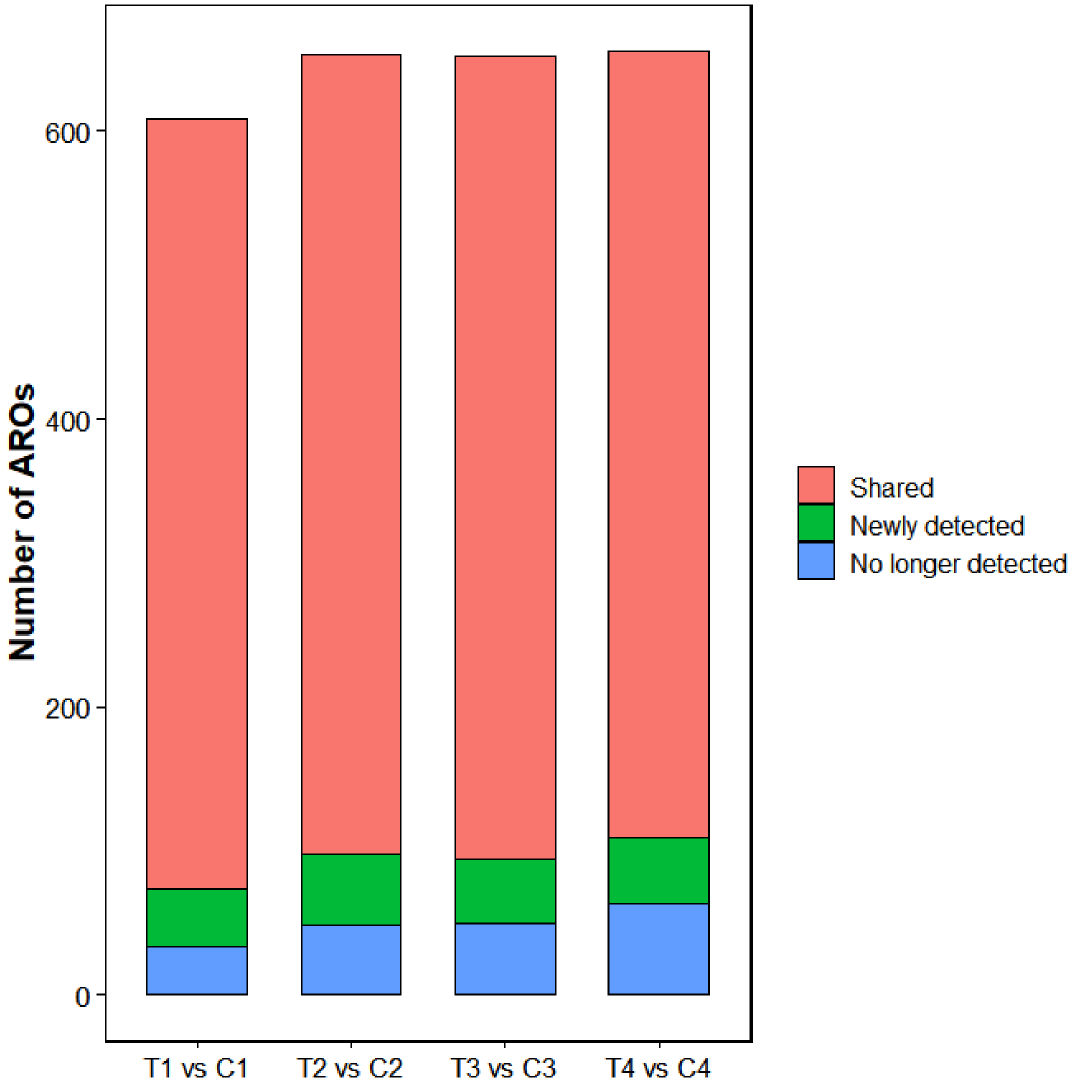

Figure S1. Visualization of ARO turnover patterns between larval treatment groups and corresponding parallel controls. Bars show the numbers of shared, newly detected and no longer detected AROs for each comparison. “Newly detected” refers to AROs detected in the larval treatment group but not in the corresponding parallel control, whereas “no longer detected” refers to AROs detected in the corresponding parallel control but not in the larval treatment group. Detection-based turnover was defined operationally and should not be interpreted as definitive acquisition or loss of ARGs.
