# Supplementary material for: Metagenomic Profiling of Antibiotic Resistance Genes in Swine Manure Residues After Sarcophaga peregrina Larval Bioconversion Under Polystyrene Particle Exposure
Source: Insects. 2026 Sep 7;17(9):936. doi: 10.3390/insects17090936 (PMC13607703; doi:10.3390/insects17090936)
Supplement: Supplementary file 1 [file insects-17-00936-s001.zip › Supplementary Table S2.pdf]

Table S2. Summary of CARD annotation and alignment information

Item	Value	Description
CARD database	CARD v3.0.9	Confirmed from the archived sequencing report.
Alignment software	DIAMOND v0.8.35	Confirmed from the archived sequencing report.
CARD alignment records	52,015	Number of rows in Supplementary Data S1.
Unique query genes with CARD hits	52,015	Unique query gene IDs in the CARD alignment table.
Annotated AROs before filtering	805	Unique ARO accessions detected before downstream filtering.
Retained AROs after filtering	692	ARO detected in ≥2 of 3 replicates in at least one group and total reads ≥100 across all samples.
Removed low-occurrence/low-read AROs	113	805 initial AROs minus 692 retained AROs.
E-value information	0.00E+00 to 1.00E-05	The retained CARD alignment table contains E-values ≤ 1e−5.
Identity (%)	37.95 ± 11.35; median 35.40; range 18.50–100.00	Summary across CARD alignment records.
Similarity (%)	58.62 ± 9.97; median 56.90; range 35.00–100.00	Summary across CARD alignment records.
Alignment length	210.9 ± 145.9; median 181.0; range 21.0–1463.0	HSP/alignment length in the CARD alignment table.
Query coverage (%)	82.22 ± 20.06; median 90.43; range 5.08–100.00	Calculated from query alignment coordinates.
Hit coverage (%)	45.93 ± 28.04; median 36.90; range 2.19–100.00	Calculated from hit alignment coordinates.
Full alignment and annotation details	Supplementary Data S1	Includes score, E-value, identity, similarity, alignment length, query/hit coverage, ARO annotation and resistance categories.

Note: Detailed CARD alignment and annotation records are provided in Supplementary Data S1. Query and hit coverage were calculated from alignment coordinates.
