# Supplementary material for: Metagenomic Profiling of Antibiotic Resistance Genes in Swine Manure Residues After Sarcophaga peregrina Larval Bioconversion Under Polystyrene Particle Exposure
Source: Insects. 2026 Sep 7;17(9):936. doi: 10.3390/insects17090936 (PMC13607703; doi:10.3390/insects17090936)
Supplement: Supplementary file 1 [file insects-17-00936-s001.zip › Supplementary Table S3.pdf]

Table S3. Summary of CARD annotation, ARO filtering and group-level ARG metrics

Group	PS particles (mg/kg)	Group-level detected ARO count	Total ARG abundance (RPKM)	ARG richness	Shannon diversity
C0	NA	566	38,809 ± 4,224	560.67 ± 10.79	4.808 ± 0.010
C1	0	567	43,093 ± 4,064	566.33 ± 2.52	4.859 ± 0.009
C2	0.001	603	42,876 ± 3,427	595.33 ± 4.04	4.924 ± 0.055
C3	0.01	607	50,664 ± 2,635	600.33 ± 12.66	4.956 ± 0.054
C4	0.1	609	50,819 ± 6,840	605.33 ± 7.02	4.819 ± 0.072
T1	0	575	51,100 ± 11,372	566.33 ± 43.62	4.736 ± 0.108
T2	0.001	605	61,114 ± 1,406	583.67 ± 60.12	4.866 ± 0.258
T3	0.01	603	57,255 ± 1,298	572.00 ± 40.04	4.885 ± 0.243
T4	0.1	592	57,088 ± 4,640	579.00 ± 59.56	4.898 ± 0.253

Note: Group-level detected ARO count was calculated using the criterion of reads number > 0 in at least two of three biological replicates within each group. ARG richness was calculated from the filtered ARO read-count matrix, whereas Shannon diversity was calculated from the filtered ARO-level RPKM matrix. Values represent sample-level means ± SD.
