# Supplementary material for: Metagenomic Profiling of Antibiotic Resistance Genes in Swine Manure Residues After Sarcophaga peregrina Larval Bioconversion Under Polystyrene Particle Exposure
Source: Insects. 2026 Sep 7;17(9):936. doi: 10.3390/insects17090936 (PMC13607703; doi:10.3390/insects17090936)
Supplement: Supplementary file 1 [file insects-17-00936-s001.zip › Supplementary Table S4.pdf]

Table S4. Statistical analyses for ARG metrics and resistome composition

Table S4A. Statistical analyses of main ARG metrics, T/C fold change and residue mass-adjusted relative ARG index

Response variable	Factor	df	F	*P*	Note
Total ARG abundance	Larval treatment	1,16	16.77	< 0.001	C0 excluded; log10-transformed
Total ARG abundance	PS particle concentration	3,16	2.23	0.12	C0 excluded; log10-transformed
Total ARG abundance	Larval treatment × PS particle concentration	3,16	1.5	0.25	C0 excluded; log10-transformed
ARG richness	Larval treatment	1,16	1.21	0.287	C0 excluded
ARG richness	PS particle concentration	3,16	0.61	0.621	C0 excluded
ARG richness	Larval treatment × PS particle concentration	3,16	0.2	0.898	C0 excluded
Shannon diversity	Larval treatment	1,16	0.42	0.526	C0 excluded
Shannon diversity	PS particle concentration	3,16	0.64	0.599	C0 excluded
Shannon diversity	Larval treatment × PS particle concentration	3,16	0.42	0.742	C0 excluded
T/C fold change in total ARG abundance	Welch's ANOVA	3, 4.03	39.99	0.0019	0.001 mg/kg > 0.01 mg/kg; other pairwise comparisons not significant
Residue mass-adjusted relative ARG index	Welch's ANOVA	3, 3.64	39.05	0.003	0.001 mg/kg > 0.01 mg/kg; other pairwise comparisons not significant

Table S4B. Games–Howell post hoc comparisons among PS particle concentration groups

Response variable	Pairwise comparison (mg/kg)	Mean difference	*P*
T/C fold change in total ARG abundance	0 vs 0.001	−0.240	0.536
T/C fold change in total ARG abundance	0 vs 0.01	0.056	0.98
T/C fold change in total ARG abundance	0 vs 0.1	0.062	0.977
T/C fold change in total ARG abundance	0.001 vs 0.01	0.296	0.001
T/C fold change in total ARG abundance	0.001 vs 0.1	0.303	0.053
T/C fold change in total ARG abundance	0.01 vs 0.1	0.007	0.999
Residue mass-adjusted relative ARG index	0 vs 0.001	−0.151	0.526
Residue mass-adjusted relative ARG index	0 vs 0.01	0.033	0.982
Residue mass-adjusted relative ARG index	0 vs 0.1	0.026	0.993
Residue mass-adjusted relative ARG index	0.001 vs 0.01	0.184	0.006
Residue mass-adjusted relative ARG index	0.001 vs 0.1	0.177	0.069
Residue mass-adjusted relative ARG index	0.01 vs 0.1	−0.007	0.997

Table S4C. Two-way ANOVA and FDR correction for ARG antibiotic class and resistance mechanism abundance

Factor	Response variable	Category	Total proportion (%)	F	*P*	FDR-adjusted *P* value
Larval treatment (F1,16)	Antibiotic class abundance	Multidrug	30.94	16.91	< 0.001	0.005
	Antibiotic class abundance	MLS	17.60	8.35	0.011	0.040
	Antibiotic class abundance	Glycopeptide	10.98	6.62	0.020	0.044
	Antibiotic class abundance	Tetracycline	10.67	2.31	0.148	0.247
	Antibiotic class abundance	Peptide	7.97	6.82	0.019	0.044
	Antibiotic class abundance	Aminoglycoside	4.81	16.91	< 0.001	0.005
	Antibiotic class abundance	Fluoroquinolone	3.16	0.51	0.485	0.623
	Antibiotic class abundance	Aminocoumarin	2.86	4.99	0.040	0.075

	Antibiotic class abundance	Phenicol	2.72	0.46	0.505	0.632
	Antibiotic class abundance	Beta-lactam	1.61	0.14	0.710	0.820
	Antibiotic class abundance	Mupirocin	1.36	15.82	0.001	0.005
	Antibiotic class abundance	Pleuromutilin	1.33	6.68	0.020	0.044
	Antibiotic class abundance	Fosfomycin	1.22	0.05	0.827	0.886
	Antibiotic class abundance	Diaminopyrimidine	1.17	1.05	0.320	0.480
	Antibiotic class abundance	Sulfonamide	0.68	0.00	0.987	0.987
	Resistance mechanism abundance	Antibiotic efflux	52.58	15.50	0.001	0.011
	Resistance mechanism abundance	Antibiotic target alteration	21.71	3.33	0.087	0.130
	Resistance mechanism abundance	Antibiotic target protection	12.06	2.61	0.126	0.162
	Resistance mechanism abundance	Antibiotic inactivation	9.56	8.03	0.012	0.036
	Resistance mechanism abundance	Antibiotic target replacement	2.51	4.69	0.046	0.103
	Resistance mechanism abundance	Antibiotic target alteration;antibiotic target replacement	0.76	0.05	0.828	0.828
	Resistance mechanism abundance	Antibiotic efflux;antibiotic target alteration	0.45	0.14	0.712	0.800
	Resistance mechanism abundance	Antibiotic efflux;reduced permeability to antibiotic	0.33	9.73	0.007	0.030
	Resistance mechanism abundance	Reduced permeability to antibiotic	0.05	4.13	0.059	0.106
	Antibiotic class abundance	Multidrug	30.94	1.90	0.171	0.443
	Antibiotic class abundance	MLS	17.60	1.92	0.168	0.443
	Antibiotic class abundance	Glycopeptide	10.98	1.73	0.200	0.443
	Antibiotic class abundance	Tetracycline	10.67	1.60	0.228	0.443
Interaction	Antibiotic class abundance	Peptide	7.97	2.51	0.095	0.443
(F3,16)	Antibiotic class abundance	Aminoglycoside	4.81	0.49	0.697	0.706
	Antibiotic class abundance	Fluoroquinolone	3.16	2.38	0.108	0.443
	Antibiotic class abundance	Aminocoumarin	2.86	1.52	0.247	0.443
	Antibiotic class abundance	Phenicol	2.72	1.45	0.266	0.443
	Antibiotic class abundance	Beta-lactam	1.61	0.47	0.706	0.706

	Antibiotic class abundance	Mupirocin	1.36	1.07	0.389	0.584
	Antibiotic class abundance	Pleuromutilin	1.33	0.78	0.523	0.685
	Antibiotic class abundance	Fosfomycin	1.22	1.63	0.223	0.443
	Antibiotic class abundance	Diaminopyrimidine	1.17	0.65	0.593	0.685
	Antibiotic class abundance	Sulfonamide	0.68	0.65	0.594	0.685
	Resistance mechanism abundance	Antibiotic efflux	52.58	2.02	0.152	0.470
	Resistance mechanism abundance	Antibiotic target alteration	21.71	1.43	0.272	0.470
	Resistance mechanism abundance	Antibiotic target protection	12.06	1.63	0.221	0.470
	Resistance mechanism abundance	Antibiotic inactivation	9.56	0.39	0.760	0.855
	Resistance mechanism abundance	Antibiotic target replacement	2.51	1.39	0.282	0.470
	Resistance mechanism abundance	Antibiotic target alteration;antibiotic target replacement	0.76	0.50	0.685	0.855
	Resistance mechanism abundance	Antibiotic efflux;antibiotic target alteration	0.45	0.17	0.912	0.912
	Resistance mechanism abundance	Antibiotic efflux;reduced permeability to antibiotic	0.33	4.60	0.017	0.150
	Resistance mechanism abundance	Reduced permeability to antibiotic	0.05	1.29	0.313	0.470
PS particles (F3,16)	Antibiotic class abundance	Multidrug	30.94	4.27	0.022	0.213
	Antibiotic class abundance	MLS	17.60	0.32	0.810	0.868
	Antibiotic class abundance	Glycopeptide	10.98	0.61	0.617	0.732
	Antibiotic class abundance	Tetracycline	10.67	0.07	0.976	0.976
	Antibiotic class abundance	Peptide	7.97	1.14	0.363	0.545
	Antibiotic class abundance	Aminoglycoside	4.81	3.09	0.057	0.212
	Antibiotic class abundance	Fluoroquinolone	3.16	1.65	0.218	0.401
	Antibiotic class abundance	Aminocoumarin	2.86	1.57	0.236	0.401
	Antibiotic class abundance	Phenicol	2.72	3.22	0.051	0.213
	Antibiotic class abundance	Beta-lactam	1.61	1.66	0.215	0.401
	Antibiotic class abundance	Mupirocin	1.36	0.58	0.635	0.732
	Antibiotic class abundance	Pleuromutilin	1.33	1.55	0.241	0.401

Antibiotic class abundance	Fosfomycin	1.22	1.88	0.174	0.401
Antibiotic class abundance	Diaminopyrimidine	1.17	3.33	0.046	0.213
Antibiotic class abundance	Sulfonamide	0.68	0.61	0.617	0.732
Resistance mechanism abundance	Antibiotic efflux	52.58	3.76	0.032	0.105
Resistance mechanism abundance	Antibiotic target alteration	21.71	0.18	0.909	0.989
Resistance mechanism abundance	Antibiotic target protection	12.06	0.04	0.989	0.989
Resistance mechanism abundance	Antibiotic inactivation	9.56	3.74	0.033	0.105
Resistance mechanism abundance	Antibiotic target replacement	2.51	3.14	0.054	0.105
Resistance mechanism abundance	Antibiotic target alteration;antibiotic target replacement	0.76	1.98	0.158	0.237
Resistance mechanism abundance	Antibiotic efflux;antibiotic target alteration	0.45	0.67	0.585	0.752
Resistance mechanism abundance	Antibiotic efflux;reduced permeability to antibiotic	0.33	3.06	0.058	0.105
Resistance mechanism abundance	Reduced permeability to antibiotic	0.05	3.35	0.045	0.105

Table S4D. PERMANOVA results based on Bray–Curtis dissimilarities of the filtered ARO-level RPKM matrix

Dataset	Factor / comparison	df	R^2	F	*P*
All samples	Treatment type: initial manure background, parallel control and larval treatment	2	0.313	5.471	0.001
C0 excluded	Larval treatment	1	0.237	8.004	0.001
C0 excluded	PS particle concentration	3	0.154	1.732	0.083
C0 excluded	Larval treatment × PS particle concentration	3	0.136	1.538	0.139

Table S4E. PERMDISP tests for differences in within-group dispersion

Dataset	Grouping variable	F	*P*
All samples	Treatment type: initial manure background, parallel control and larval treatment	11.09	0.001
C0 excluded	Parallel control vs larval treatment	7.51	0.007
C0 excluded	Groups C1–C4 and T1–T4	3.7	0.012

Note: C0 was excluded from factorial ANOVA, Welch's ANOVA, Games–Howell post hoc tests and two-factor PERMANOVA. Benjamini–Hochberg FDR

correction was applied separately to antibiotic class and resistance mechanism abundance within each model factor. Categories with raw $P < 0.05$ but FDR-adjusted $P \geq 0.05$ were interpreted as nominally significant only. PERMANOVA and PERMDISP tests were based on Bray–Curtis dissimilarities with 999 permutations. The T/C fold change and residue mass-adjusted relative ARG index were compared among PS particle concentration groups using Welch's ANOVA followed by Games–Howell post hoc tests.
